# Ophthalmic manifestations are associated with reduced tear lymphotoxin-α levels in chronic ocular graft-versus-host disease

**DOI:** 10.1186/s12886-022-02251-y

**Published:** 2022-01-10

**Authors:** Jiao Ma, Chendi Li, Yinghan Zhao, Zhan Shen, Bohao Hu, Rongmei Peng, Jing Hong

**Affiliations:** 1 Department of Ophthalmology, Peking University Third Hospital, No.49 Garden North Road, Haidian, Beijing, 100191 China; 2 Beijing Key Laboratory of Restoration of Damaged Ocular Nerve, Peking University Third Hospital, Beijing, China

**Keywords:** Biomarker, Lymphotoxin-α, Ocular graft-versus-host disease, Tear film

## Abstract

**Purpose:**

To determine the role tear lymphotoxin-α (LT-α) in chronic ocular graft-versus-host disease (oGVHD).

**Methods:**

Twenty-two chronic oGVHD and 17 control tear samples were collected, and commercial test strips were used to detect LT-α concentrations. Concentration differences between patients with and without oGVHD were determined via Mann-Whitney *U* test. The correlation between LT-α levels and ophthalmic parameters was analyzed using Spearman’s test.

**Results:**

The concentration of LT-α was significantly lower in oGVHD patients than in controls. LT-α levels were significantly correlated with OSDI, NIH eye score, T-BUT, and CFS among all participants. ROC analysis revealed that the area under the curve of LT-α was 0.847, and the cutoff value for chronic oGVHD diagnosis was 0.203 ng/mL.

**Conclusion:**

Our study revealed the significant decrease of tear LT-α in oGVHD, and suggested LT-α as a promising marker for chronic oGVHD diagnosis.

## Introduction

Allogeneic hematopoietic stem cell transplantation (allo-HSCT) is a major therapy for many patients with hematological malignances [1]. However, chronic graft-versus-host disease (GVHD) represents a common complication of allo-HSCT, affecting 30–70% of human leukocyte antigen-matched recipients [2, 3]. As an immune disease involving tissue inflammation and fibrosis, chronic GVHD may lead to permanent dysfunction in multiple organs. Notably, up to 60–90% of chronic GVHD patients suffer from ocular GVHD (oGVHD) [4], characterized by new-onset dry eyes, keratoconjunctivitis sicca, cicatricial conjunctivitis, and confluent areas of punctate keratopathy [5]. Recently, we and others have reported the significant elevation of the levels of several proinflammatory cytokines (such as IL-1β, IL-2, IL-6, IL-8 IL-7, IL-10, IFN-γ and TNF-α) in chronic oGVHD tear films [6–9], suggesting a correlation between elevated proinflammatory cytokine levels and increased inflammation in oGVHD pathogenesis.

Lymphotoxin-alpha (LT-α, formerly named TNF-β) is a TNF superfamily cytokine that plays a specific role in the development and orchestration of immune responses [10]. The expression of LT-α is restricted to activated CD4+ Th1 and Th17 subsets, CD8+ T cells, B cells, and natural killer cells [11–13], all of which are closely implicated in GVHD. Increasing evidence supports that LT-α is vital in systemic GVHD pathogenesis. Markey KA and colleagues used multiple preclinical GVHD models and suggested that LT-α is an important contributor to GVHD [14]. Chiang EY et al. reported that LT-α expression was upregulated in activated human donor lymphocytes and that targeted depletion of these donor cells could ameliorate GVHD [15]. These studies revealed the crucial effect of LT-α in GVHD pathogenesis and suggested the potential application of LT-α antagonists for GVHD treatment; however, the role of LT-α in oGVHD remains unclear.

To reveal the role of LT-α in oGVHD, we recruited HSCT recipients with and without chronic oGVHD and determined LT-α levels in their fresh tears using colloidal gold immunochromatography strips. We then calculated the correlation of LT-α levels with ocular surface parameters and further evaluated the diagnostic value of tear LT-α for oGVHD.

## Materials and methods

### Ethics and clinical subjects

This study was approved by the Peking University Third Hospital Medical Science Research Ethics Committee and performed in accordance with the tenets of the Declaration of Helsinki. Written informed consent was obtained from all the subjects after explaining the nature and possible consequences of the study.

Chronic oGVHD patients were diagnosed in accordance with the National Institutes of Health (NIH) consensus [5], and inclusion criteria were set as we previously described [16]: (1) NIH eye score ≥ 1 point; (2) ocular surface disease index (OSDI) score > 12 points; (3) corneal fluorescein staining (CFS) ≥ 1; and (4) Schirmer’s test without topical anesthesia ≤5 mm in 5 min. Exclusion criteria included ocular surgery within the past 6 months, ocular injury, other ocular diseases (such as infection, allergy, glaucoma, retinopathy, and autoimmune disease), pregnancy, and long-term use of any topical ocular medications.

### Tear collection and LT-α analysis

Tear collection was performed as we previously described [16]. First, 30 μL of a sterile normal saline solution was dropped into the conjunctival sac, and then diluted tear samples were collected with a capillary tear collector. To immediately determine the tear LT-α concentration, a commercial test strip based on a colloidal gold and immunochromatography assay (S02A, Seinda Biomedical Corporation, Guangdong, China) was utilized following the instructions. Briefly, a 2.2 μL tear sample was added to the sampling hole, three drops of the diluent were added to the diluting hole, the strip was then inserted into the analyzer (S03A, Seinda Biomedical Corporation), and the LT-α level was registered 15 min later.

### Clinical evaluation

Clinical examinations were always performed by the same clinician. First, the OSDI questionnaire was administered to assess ocular symptoms over the preceding week. Then, tear samples were collected, and LT-α levels were immediately determined as we described above. Next, best corrected visual acuity and intraocular pressure were detected; fluorescein tear film break-up time (T-BUT), CFS score and NIH eye score were then evaluated in the clinic. Finally, Schirmer’s test without anesthesia was conducted using the commercial strips.

### Statistical analysis

Statistical analysis and graphing were performed via GraphPad Prism 8.0 software (GraphPad Software, Inc., San Diego, CA, USA). The normality assumption was validated with the Shapiro-Wilk test. For quantitative variables, normally distributed data were analyzed with Student’s *t*-test and presented as the mean ± standard deviation; nonnormally distributed data were analyzed with the Mann-Whitney *U* test and presented as the median ± interquartile range (IQR). For qualitative data, the chi-square test was used to assess the association. Spearman’s test was used to analyze the correlation of the LT-α concentration with ocular surface parameters. The results were considered statistically significant if the *P* value was less than 0.05.

## Results

### Demographic and clinical details of the participants

Clinical and demographic characteristics are presented in Table 1. Statistical analysis confirmed that there were no significant differences in age (*P* = 0.173) or sex (*P* = 0.092) between patients with and without oGVHD.

**Table 1:** Patient characteristics

Characteristics	oGVHD (n=22)	Control (n=17)	*P* value
Age, years1	31.52 ± 9.63	26.86 ± 9.86	0.173
Gender2			0.092
Male, n (%)	11 (50.0%)	13 (76.47%)	
Female, n (%)	11 (50.0%)	4 (23.53%)	
Hematologic diagnosis, n (%)			0.711
AML (acute myelogenous leukemia)	15 (68.2%)	10 (58.8%)	
ALL (acute lymphoblastic leukemia)	4 (18.2%)	5 (29.4%)	
MDS (myelodysplastic syndromes)	3 (13.6%)	2 (11.8%)	
HLA matching			0.291
Haploidentical	18 (81.8%)	15 (88.2%)	
Non-identical	0	1 (5.9%)	
Identical	4 (18.2%)	1 (5.9%)	
Other current organ involvement in cGVHD			0.073
Gastrointestinal tract	2 (9.1%)	2 (11.8%)	
Lung	4 (18.2%)	1 (6.2%)	
Liver	9 (40.9%)	0	
Oral cavity	19 (86.4%)	1 (6.2%)	
Skin	20 (90.9%)	7 (41.2%)	
Current systemic immunosuppressive treatment			0.827
None	6 (27.3%)	5 (29.4%)	
Calcineurin inhibitor	4 (18.2%)	3 (17.6%)	
Steroid	5 (22.7%)	2 (11.8%)	
Calcineurin inhibitor + steroid	7 (31.8%)	7 (41.2%)	
Period after HSCT, months	19 ± 19	15 ± 11	0.084

Mean values ± SD, median ± IQR, or percentages were shown as above. There were no significant differences in age1 (*P* = 0.173) or gender2 (*P* = 0.092) between oGVHD patients (*n*=22) and controls (*n*=17)

Ocular details were summarized in Table 2. There were significant differences in OSDI score, NIH eye score, CFS, T-BUT, and Schirmer’s test between oGVHD patients and the controls (*P* < 0.0001 for all), and the best corrected visual acuity of oGVHD patients was significantly poorer than that of the controls (*P* = 0.035).

**Table 2:** Ocular parameters of patients with and without oGVHD

Ocular parameters	oGVHD (***n***=22)	Control (***n***=17)	***P*** value
OSDI score	37.25 ± 20.81	1.60 ± 1.58	<0.0001****
NIH eye score	2 ± 2	0 ± 0	<0.0001****
Corneal fluorescein score	10 ± 7.75	0 ± 0	<0.0001****
Fluorescein tear film break-up time (s)	2 ± 1.25	10 ± 2	<0.0001****
Schirmer’s tear secretion score (mm)	1 ± 2.5	16.5 ± 8.5	<0.0001****
Best corrected visual acuity (logMAR)	0.43 ± 0.41	0.19 ± 0.22	0.035*
Intraocular pressure (mmHg)	13.0 ± 3.4	15.1 ± 4.2	0.163

Ocular parameters were compared between patients with (*n*=22) and without (*n*=17) oGVHD. Data were presented as mean ± SD or median ± IQR. *P<0.05, **P<0.01, ***P<0.001, ****P<0.0001

### Tear LT-α concentration

**Fig. 1:**
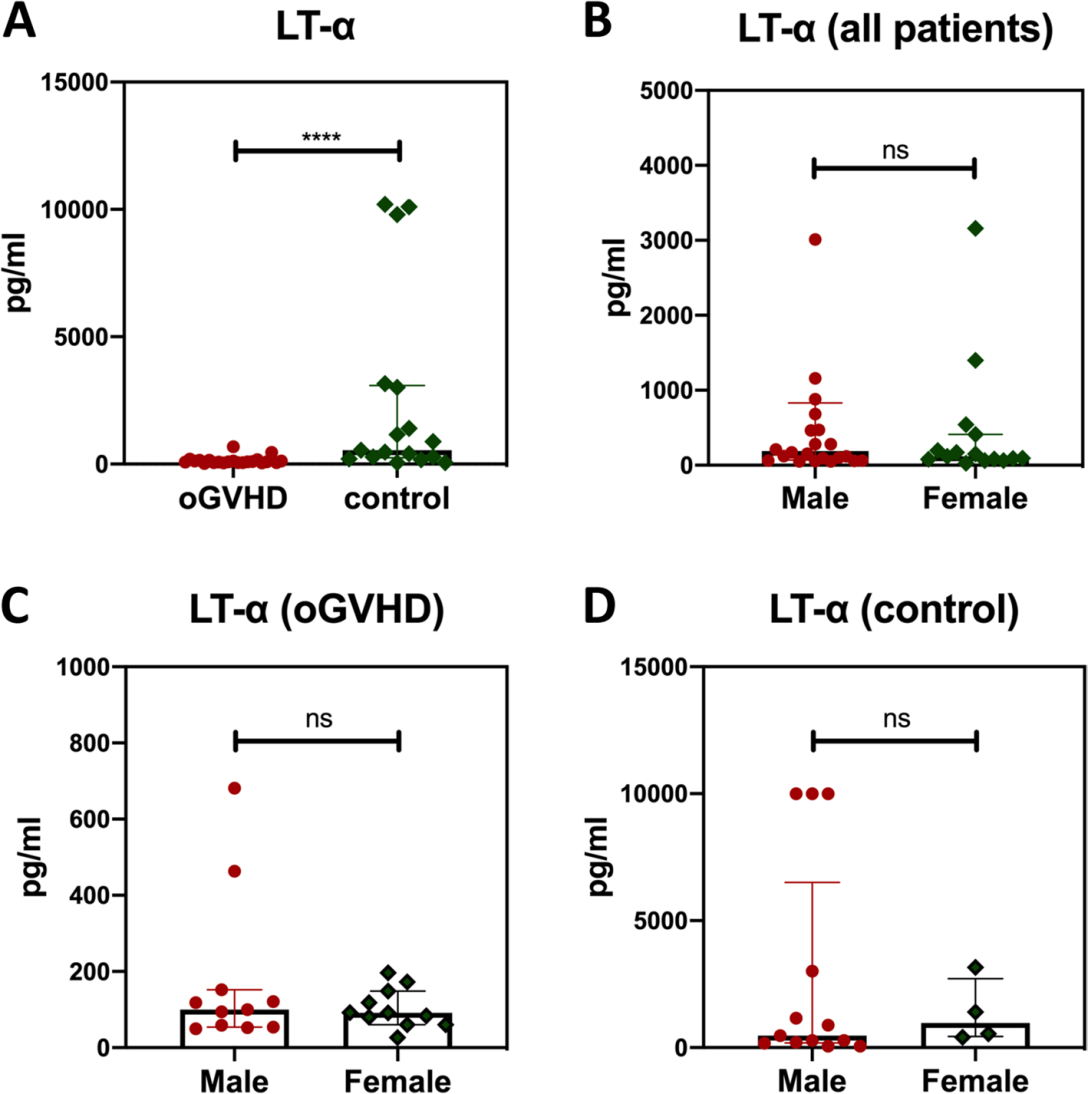
Tear LT-α concentration. **A** Tear concentration of LT-α was compared between patients with (*n* = 22) and without (*n* = 17) oGVHD. **B-D** Tear LT-α levels were compared between the male and female among all patients **(B)**, oGVHD subpopulations (**C**), and controls (**D**). *P < 0.05, **P < 0.01, ***P < 0.001, ****P < 0.0001 using Mann-Whitney *U* test

The concentration of LT-α in tears was compared between patients with (*n* = 22) and without chronic oGVHD (*n* = 17), and interestingly, we observed a significant decrease in LT-α concentration in oGVHD patients (0.093 ± 0.090 ng/mL) compared to that of controls (0.54 ± 2.84 ng/mL) (median ratio = 5.8, *P* < 0.0001, Fig. 1A). To determine if tear LT-α levels would be influenced by sex differences, we then compared LT-α concentration between the male and female among all patients, oGVHD subpopulations, and controls, and the analysis results revealed no significant differences among all of them (*P* > 0.05 for all, Fig. 1B-D).

### Correlation and ROC analysis of LT-α levels

The correlation between tear LT-α levels and ocular surface parameters was then assessed in all participants and oGVHD subpopulations (Table 3). Among all participants, tear LT-α levels correlated positively with T-BUT (*r* = 0.607) and negatively with OSDI score (*r* = − 0.565), NIH eye score (*r* = − 0.628), and CFS (*r* = − 0.608) (*P* < 0.001 for all), whereas in oGVHD subpopulations, LT-α level exhibited no significant correlations with all these ocular surface parameters. These results suggested tear LT-α as a potential marker for the diagnosis but not severity assessment of oGVHD.

**Table 3:** Correlation analysis between tear LT-α and clinical parameters

Ocular surface parameters	All patients (rho, P)	oGVHD patients (rho, P)
OSDI score	-0.565, 0.0008***	-0.120, 0.594
NIH eye score	-0.628, <0.0001****	-0.158, 0.482
Corneal fluorescein score	-0.608, <0.0001****	-0.278, 0.211
Fluorescein tear film break-up time (s)	0.607, <0.0001****	0.277, 0.212

Correlation analysis was calculated via Spearman’s test. P value less than 0.05 was considered significant, *P<0.05, **P<0.01, ***P<0.001, ****P<0.0001; rho, Spearman ranked correlation coefficient

**Fig. 2:**
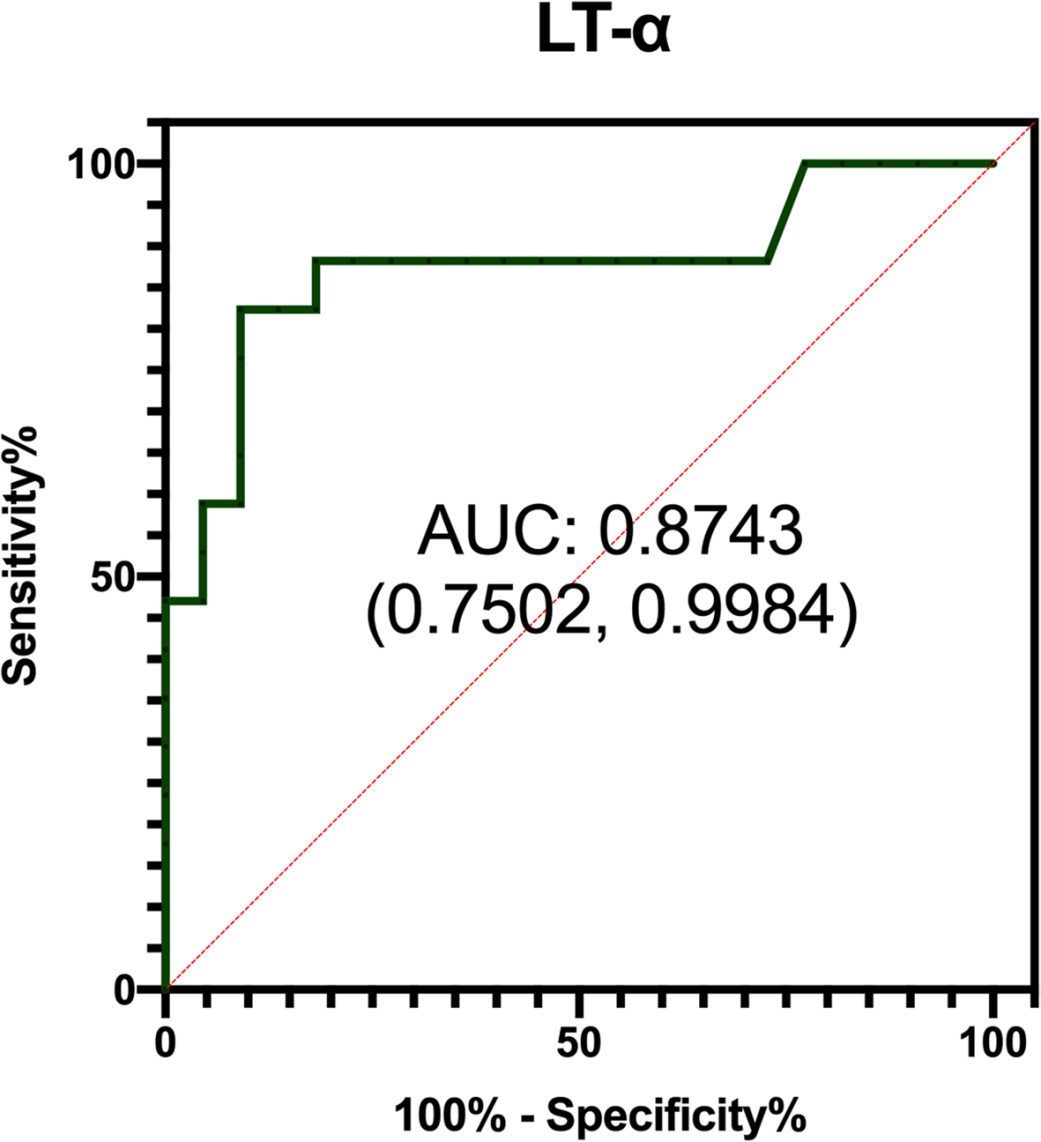
ROC curve of LT-α. Receiver operating characteristic (ROC) curve of tear LT-α to predict oGVHD. Area under the curve (AUC) = 0.8743; 95% CI (0.7502 to 0.9984); *P* < 0.0001

We next performed ROC analysis to further evaluate the cutoff value of tear LT-α levels for predicting oGVHD diagnosis (Fig. 2). The area under the curve (AUC) of the LT-α concentration was 0.874 (*P* < 0.0001), and the cutoff value of LT-α concentration was 0.203 ng/mL (sensitivity 82.4%, specificity 90.91%).

## Discussion

LT-α is a cytotoxic cytokine that belongs to the TNF superfamily and is predominantly produced by lymphocytes [17]. Similar to TNF, LT-α can promote T cell function and contribute to various inflammatory responses [18]. Increasing findings support that upregulated LT-α expression plays a vital role in immune diseases and that blocking LT-α might be a potential treatment for diseases such as rheumatoid arthritis and GVHD [14, 15, 19]. However, in the present study, we observed a decreased level of tear LT-α in oGVHD patients compared with that in controls, which implied that LT-α might not simply act as a proinflammatory cytokine as previous studies have reported. In addition, we noticed that the tear LT-α level was inversely correlated with ophthalmic symptoms, suggesting that LT-α might also have a regulatory effect on chronic oGVHD. Moreover, the AUC of the LT-α concentration was 0.874, and the cutoff value of LT-α concentration was 0.203 ng/mL. These results indicated that tear LT-α levels lower than 0.203 ng/mL could be useful to predict the presence of chronic oGVHD.

Dry eye disease (DED) or keratoconjunctivitis sicca represents the most common manifestation of chronic oGVHD. Interestingly, a recent study enrolled 782 dry eye patients and 306 non-dry eye controls and reported that the tear LT-α level was significantly lower in dry eye patients than in controls [20], consistent with our results observed in oGVHD patients. Therefore, we speculated that LT-α might exert a similar influence on DED and oGVHD. Another study based on DED patients revealed that tear cytokine expression was distinct between patients with high LT-α levels and patients with low LT-α levels [21], indicating a different pathogenesis between high- and low-LT-α DED patients. In this study, we noticed a low tear LT-α level in most oGVHD patients, whereas some oGVHD patients exhibited a much higher level of tear LT-α. Based on this finding, a comparison study on oGVHD patients with different LT-α levels may reveal new insights into oGVHD mechanisms. Animal models with LT-α depletion may also provide an interesting future direction to explore the role of LT-α during the oGVHD initial and developmental stages.

## Conclusions

Our study confirmed the diagnostic value of tear LT-α for chronic oGVHD. Consistent with the results in DED, we also found that the tear LT-α level was significantly decreased in chronic oGVHD, suggesting that LT-α might exhibit a regulatory effect on oGVHD-related dry eye symptoms.

## Data Availability

The datasets used and/or analyzed during the current study are available from the corresponding author on reasonable request.

## Abbreviations

Allo-HSCT: Allogeneic hematopoietic stem cell transplantation
AUC: Area under the curve
CFS: Corneal fluorescein staining
DED: Dry eye disease
GVHD: Graft-versus-host disease
IQR: Interquartile range
LT-α: Lymphotoxin-α
NIH: The National Institutes of Health
oGVHD: Ocular GVHD
OSDI: Ocular surface disease index
ROC: Receiver operating characteristic
T-BUT: Tear film break-up time
